# The American Medical Association and Medical Education

**Published:** 1875-05

**Authors:** 


### Editorial.

### The American Medical Association and Medical Education.

From what we can learn from those who were present, and from the reports which have reached us, we are inclined to the opinion that the meeting at Louisville was a successful one. The address by the President and by the Chairmen of the different sections were of a high order, and as a whole, will meet with general approbation. Dr. Bowling in his address referred to the much vexed question of medical education, and the preliminary examination of young men intending to study medicine. He said:—If this body has not of itself accomplished all its friends hoped for in the beginning, in elevating the standard of medical education, they must be satisfied to know that that standard, notwithstanding, has been regularly going up, fully abreast with the progress of our new country in every other department of human learning and in all the arts and appliances of a rapidly developing civilization. The spring can only well up the waters sent to it, purifying them in the process, and the sea is but the representative of many waters. The schools must take such material as they can get and make the most of it; and the American Medical Association as in the past, so now and hereafter, is obliged to consist of such representative medical men as the schools send it. In order to make the material sent to the schools of a better order he made the following proposition:—"Let it be solemnly resolved by this meeting that it shall be regarded as derogatory to the character of any physician, in any part of the United States, to take under his charge as a student of medicine any one who cannot exhibit evidence of having taken a degree in a regularly chartered college or a certificate of qualifications necessary to become a student of medicine, from a board of examiners appointed for the purpose by the American Medical Association." It appears to us that this is somewhat out side of the prerogatives of the Association, all that it can do is to urge upon State Societies the importance of this step and to show them the advisability of following the example set by the New York State Medical Society and some others. The importance of this step is evident to all, but its details should be faithfully and carefully carried out, it will not do to appoint a board which will give a young man a certificate of proper qualifications as a student of medicine, merely because he evinced a willingness to come before the board, without asking him a question. A preliminary examination will deter no young man who is in earnest from entering the ranks of the medical profession, and all others had far better remain outside. Whether it be called a burst of centennial patriotism or a blindness to the faults of the American "*system*" of medical education, we are forced to say; that in our country and under our system, imperfect as it is, there are constantly springing up men, who, for real worth and solid information, we will willingly compare with many European physicians who have a line of initials following their names, which nearly includes the alphabet.

In contrast with the remarks of Dr. Bowling was the address of Dr. Edgar before the Association of Medical Editors, at the Galt House, on Monday evening. He took for his subject, medical advertising. In the course of his remarks he made extended allusion to Medical Education, referring to the final rather than to the preliminary examination. He spoke of the ease in which medical schools are organized, and their employment as advertising mediums for the members of the faculty, deploring the fact that so many schools made it so easy to obtain a degree.

The remedy which Dr. Edgar proposes is a good one and one which we are heartily in favor of, if it can be properly accomplished, it is the establishment of State Boards of Medical Examiners, a diploma from which shall be necessary for the practice of medicine. But there stands in the way of the accomplishment of this greatly desired reform many obstacles, among which however we are not so willing as Dr. Edgar, to class those connected with the schools except it be those established for private and unworthy motives, the result of petty professional jealousies and rivalries. The conditions of society and government are widely different here from those in Europe. There no school or "pathy" is recognized, but all alike are required to pass the same examination. In this country where freedom of thought and action is universal, a different state of things exists and the Homeopath and Eclectic *et id omne genus* would naturally object to appearing before a board composed of regular physicians and *vice versa.* For these and other objections however there is a remedy which will doubtless present itself in time, but the time does not seem to have arrived. The people as well as the profession, must be convinced of the advisability of such a move, and must learn the difference between a true doctor and a mere pretender. Until the good time shall come, that long desired millenium of the medical profession when peace and harmony shall reign in its ranks, when the people shall cease to put their trust in magnetic healers and clairvoyant charlatans, the advice which we have to offer is summed up in the few words which one older and more experienced in the various phases of medical education, gave as his advice. "*Let every physician cease to watch his neighbor and concern himself about the affairs of others, and turning his glance inward, study his own defects and seek by study and observation to make himself the best informed physician in his city or state or country.*" And in fact herein lies the solution of the whole matter. The advancement of medical education in America lies not in Associations but in individuals, not in resolutions and preambles, but in the determination of individual members to be more worthy of the high office of physicians. When each individual member of the profession realizes this, the remedy will be natural and easy.

We are sorry to say it, but truth compells the admission, that the standard of education is not the only one which needs elevating. The moral element, the *esprit de corps* needs purifying. Petty jealousies and discords in professional ranks are of deplorable frequency, and the knowledge of their occurrence often leads young men of refined and educated tastes to hesitate upon entering a profession with such a record. Nor is this all, nor is it the most deplorable feature of the case. Physicians are found who will openly resort to the tricks of the imposter and charlatan, who are versed in intrigue and trickery, and who stoop to acts from which one who made but few pretentions to honesty would shrink. Unfortunate in early training some go through life unable to appreciate the motives of those infinitely above them; they impute their actions to motives as low as their own. While they are justly condemned pity should be felt toward them that they should thus be the unfortunate victims of circumstances. Warped and twisted by early misdirection they never see the beauty which is all around them. While this is often the case the sad truth remains that some, few let us hope, enter upon a dishonest course from deliberate choice, dead weights which drag down and defile the noble profession under whose cloak they seek to hide. We have drawn the picture in black color, but we have not done so from choice, but rather in sorrow. Its contemplation is so painful that we would gladly forget its existence.

